# Endobronchial Electrocautery Using Snare

**DOI:** 10.1155/DTE.2.207

**Published:** 1996

**Authors:** Masaaki Kawahara, Kiyoyuki Furuse, Nagahisa Kodama, Mitsumasa Ogawara, Shinji Atagi, Takao Kamimori, Mitsunobu Nakao, Nobuyuki Naka

**Affiliations:** National Kinki Central Hospital for Chest Diseases 1180 Nagasme Osaka Sakai 591 Japan

## Abstract

Between May 1987 and March 1994, upper airway and tracheobronchial electrosurgery with snare was performed in 13 patients (10 men and 3 women), ranging in age from 18 to 87 years. Four patients had benign lesions, and nine had malignant tumors. Total eradication has been achieved in the two patients with benign lesions. Electroexcision of the endobronchial portion of the tumor helped to clear the respiratory airways in all cases with malignant tumors. There has been no major side effects such as bleeding due to this method. Electrocautery is an available economical tool, which helps to diagnose and treat obstructing airway mass lesions.


*Diagnostic and Therapeutic Endoscopy*, 1996, Vol. 2, pp. 207–210
Reprints available directly from the publisher
Photocopying permitted by license only

© 1996 OPA (Overseas Publishers Association)
Amsterdam B. V. Published in The Netherlands
by Harwood Academic Publishers GmbH
Printed in Singapore

### Endobronchial Electrocautery Using Snare

MASAAKI KAWAHARA, KIYOYUKI FURUSE, NAGAHISA KODAMA, MITSUMASA OGAWARA, SHINJI ATAGI, TAKAO KAMIMORI, MITSUNOBU NAKAO, and NOBUYUKI NAKA

*National Kinki Central Hospital for Chest Diseases, Osaka, Japan*

*(Received May 22, 1995; in final form October 26, 1995)*

Between May 1987 and March 1994, upper airway and tracheobronchial electrosurgery with snare was performed in 13 patients (10 men and 3 women), ranging in age from 18 to 87 years. Four patients had benign lesions, and nine had malignant tumors. Total eradication has been achieved in the two patients with benign lesions. Electroexcision of the endobronchial portion of the tumor helped to clear the respiratory airways in all cases with malignant tumors. There has been no major side effects such as bleeding due to this method. Electrocautery is an available economical tool, which helps to diagnose and treat obstructing airway mass lesions.

**KEY WORDS:** Lung neoplasms, electrocoagulation, wire snare, bronchoscopy, granuloma, airway obstruction

### INTRODUCTION

Electrocautery using a snare loop is a valuable surgical and endoscopic tool with the potential for significant upper and lower airway applications, as well as for the gastrointestinal tract. Experience with endobronchial electrocautery is limited to a few observations (1–3), mainly because of the recent availability of laser therapy. However, laser therapy takes much more time to evaporate obstructing endobronchial lesions and produces more gas than electrocautery with snare does. In this article, we report on 13 cases of electrocautery with snare applied through a fiberoptic bronchoscope to obstructing upper airway and tracheobronchial lesions.

### MATERIALS AND METHODS

Between May 1987 and March 1994, upper airway and tracheobronchial electrosurgery with snare was performed in 13 patients (10 men and 3 women), in age ranging from 18 to 87 years. One patient had electrosurgery twice and another four times. We used an Olympus fiberoptic bronchoscope (BF-1T-10 or 1T-30), diathermic adult snares (Olympus no. SD-5L), and a high-frequency electrosurgical unit (Mera MS-5500N). All patients received local anesthesia with a transoral fiberoptic bronchoscope. We did not use a rigid bronchoscope. Premedication included 0.4 mg of atropine sulfate and 4 mg of oxycodone hydrochloride 30 min before bronchoscopy. A plate electrode was applied to the patient's thigh. An endotracheal tube was used in all patients with tracheobronchial lesions. The diathermic snare was passed via the fiberoptic bronchoscope with an instrument canal, and the tumor was grasped by the wire loop snare. Electrocrossing of tissue was accomplished by slowly drawing the wire snare into the catherter. A blended current of cutting and coagulation was used for the earlier patients and a coagulation alone was used for the recent patients. After electrocrossing, the tumor was grasped with the forceps for the removal of foreign bodies and extracted. If the dissected mass was too large to pass through the endotracheal tube, it was grasped by the forceps and extracted with the endotracheal tube. The duration of electrocautery with the diathermic snare was within 30 secs.

### CASE REPORTS

### Case 3

A 58-year-old woman had been operated on for renal cell carcinoma 3 years before this procedure. She had a polypoid tumor at the right truncus superior, which had

Address for correspondence: Masaaki Kawahara, M.D., National Kinki Central Hospital for Chest Diseases, 1180 Nagasme, Sakai Osaka 591, Japan. Tel. 0722-52-3021; Fax. 0722-51-1372.

almost completely obstructed the right main bronchus. She had complained of dyspnea on effort and a febrile episode. Her symptoms began 3 months before admission. Bronchoscopy was performed under local anesthesia. The right main bronchus was almost completely obstructed by a polypoid tumor (Fig. 1, left). A wire loop cautery snare was passed through a flexible bronchoscope. A polypoid tumor, measuring 6 mm by 8 mm in diameter, was removed without difficulty. A blended current was used. The time required for the removal was within 30 secs. The tumor was grasped with forceps for the removal of foreign bodies and extracted. Figure 1 (right) shows the patency of the right main bronchus just after electrocautery. There was no problem with bleeding or smoke. The histologic diagnosis of this tumor was endobronchial metastatic renal cell carcinoma. After the procedure, the patient had no complaints of dyspnea or fever due to the obstruction of the right main bronchus. However, the patient died of dissemination of renal cell carcinoma with a patent airway 31 months after the procedure.

### RESULTS

The results of electrocautery with snare in all patients are listed in Table 1. Complete removal of upper airway (epipharynx) and endotracheobronchial mass was possible in 3 (cases 1, 5, and 9) of 13 patients. These patients had granulomas in the epipharynx (case 1), trachea (case 5), and the right upper lobe bronchus (case 9), respectively. One patient (case 1) with pulmonary tuberculosis, who complained of hemosputum, had a granuloma in the epipharynx. This granuloma was found during bronchoscopic examination for the evaluation of hemosputum and was removed completely by electrocautery with snare. Another patient (case 5) was treated with concurrent chemoradiotherapy for squamous cell lung cancer in the right upper lobe. After the induction therapy, bronchofiberscopic examination was performed for the evaluation of tumor response to therapy. Then a polypoid mass was found in the right upper lobe bronchus. The histologic diagnosis of this polypoid mass removed by electrocautery with snare was granuloma. The other patient (case 9) with

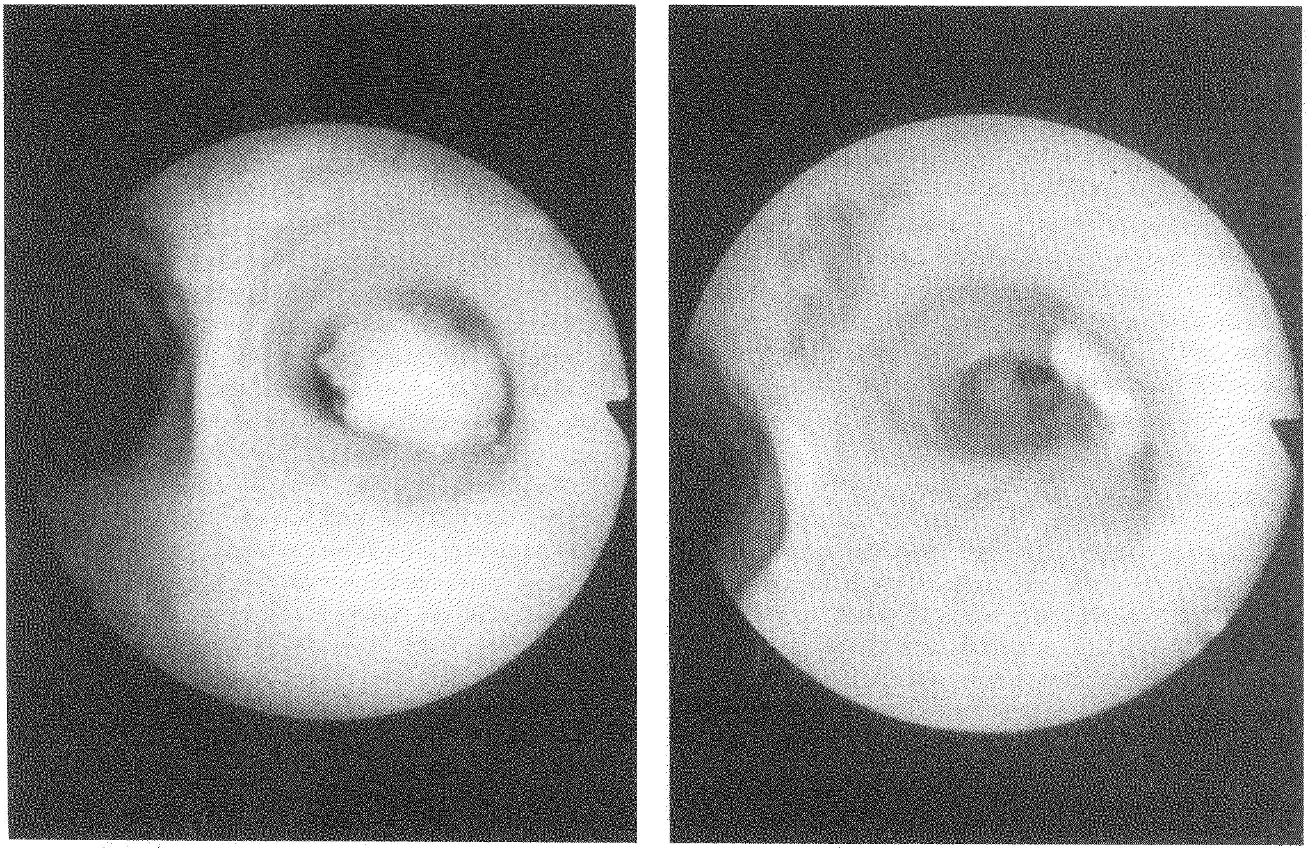

**Figure 1** Case 3. Renal cell carcinoma protruding into the right main bronchus before (left) and after (right) endobronchial electrocautery with snare.

**Table 1** Results of Electrocautery Using Snare

| *Patient* | *Age (yr.)* | *Sex* | *Histology* | *Location* | *Type of Removal* | *No. of Treatments* | *Results: Eradication of Lesion* |
|---|---|---|---|---|---|---|---|
| 1 | 18 | M | Granuloma | Epipharynx | Snare | 1 | Complete |
| 2 | 55 | F | Granuloma | Rt main br | Laser+ Snare | 1 | Partial |
| 3 | 58 | F | Renal cell ca | Rt main br | Snare | 1 | Complete |
| 4 | 70 | M | Squamous cell ca (lung) | Rt inf br | Snare | 1 | Partial |
| 5 | 50 | M | Granuloma | Rt tr sup | Snare | 1 | Complete |
| 6 | 66 | M | Colon ca | Rt main br | Laser+ Snare | 1 | Complete |
| 7 | 66 | M | Squamous cell ca (lung) | Lt main br | Snare | 1 | Partial |
| 8 | 87 | M | Squamous cell ca (lung) | Rt tr inf | Snare | 1 | Partial |
| 9 | 38 | F | Granuloma | Trachea | Laser+ Snare | 1 | Complete |
| 10 | 64 | M | Squamous cell ca (lung) | Lt main br | Snare | 2 | Partial |
| 11 | 52 | M | Large cell ca | Larynx | Snare | 1 | Complete |
| 12 | 67 | M | Squamous cell ca (lung) | Rt main br | Snare | 1 | Partial |
| 13 | 63 | M | Thyoid ca | Rt int m br | Snare | 3 | Partial |
| | | | | Rt tr inf | Snare | 1 | Complete |

Abbreviations: M,male; F,female; ca,carcinoma; rt,right; lt,left; br,bronchus; tr,truncus; inf,inferior; int m,intermediate pulmonary tuberculosis had a granuloma in the trachea, which developed after the T-tube removal. More than 75% of the endobronchial malignant tumor was removed in 10 other patients with 11 lesions. Because of tumor regrowth, one patient (case 10) required another use of the snare and another patient (case 13) needed two more snare procedures. The Cd-YAG laser was used before electrosurgery with snare in three patients (cases 2, 6, and 9). In one patient (case 6), this use of the Cd-YAG laser proved effective to debulk the polypoid tumor and to allow for the snare to go beyond the tumor and grasp it. Bronchoscopic electrosurgery with snare was not accompanied by massive hemorrhage in any cases and no other complication, such as perforation or tracheal fire, was observed.

### DISCUSSION

There are two methods used for endobronchial electrosurgery: excision of tumors with a diathermic snare, and electrodestruction of tumors with a cautery probe. As described by Gerasin and Shafirovsky (1), electrodestruction by the probe may be replaced by laser coagulation because the latter has proven to be more effective. However, for removal of polypoid lesions, laser or electrodestruction with the probe requires destruction of the whole mass by vaporization, takes more time to accomplish, generates higher thermal output, and produces more gaseous byproducts (2). Mainly, electrosurgery with a diathermic snare has been used as a palliative treatment to remove a major part of the tumor or mass and restore air passage. In addition, this procedure is also applicable for the diagnosis of a polypoid mass in the major bronchi. Although this procedure is usually used via a rigid brochoscopic tube under general anesthesia (1,4,5), we were able to effectively use this via a flexible bronchoscopic tube under local anesthesia without complications. The decision whether or not to use, rigid tube may depend on the size of tissue fragment resected.

Considering the easy availability of this method, we also applied it to the removal of tumors located in the epipharynx and larynx. In these cases, snaring the mass by the bronchoscope was more effective than by direct vision because of the magnified view of the tumor through the bronchoscope.

The duration of the effectiveness after electrocautery compared to that after laser treatment remains to be determined. This may largely depend on the characteristics of the tumor, rather than the procedure.

As for the disadvantages of electrocautery, hemorrhage, perforation, fire and difficulty in shielding the instrument have been listed (1,2,6). However, we did not experience any of these disadvantages.

Initially, a blended current of cutting and coagulation was used; however, for the most recent patients coagulation alone was used, because the coagulation mode was enough to cut the lesion with the snare. Concerning bleeding, this mode may be safer than blended current. Some failure to perform electrocautery was experienced, with the major cause being the inability to pass the snare loop beyond the polypoid tumor. In such patients, laser treatment followed by electrocautery may be useful.

In conclusion, electrocautery with snare is a readily available, economical tool that can assist in the diagnosis and therapy of obstructing airway lesions.

### ACKNOWLEDGMENTS

The authors wish to express their appreciation to Yuki Hirochi and Erina Hatashita for their assistance with the preparation of this paper.

### REFERENCES

1. Gerasin VA, Shafirovsky BB. Endobronchial electrosurgery. Chest 1988;93:270–274.
2. Hooper RG, Jackson F. Endobronchial electrocautery. Chest 1985;87:712–714.
3. Taguchi H, Nagata T, Kawai H, et al. High-frequency electrosurgical treatment of tracheal obstruction using the flexible bronchofiberscope. In: Bronchology: research, diagnostic and therapeutic aspects. The Hague: Martinus Nijhoff Publishers, 1981:563–565.
4. Spinelli P, Pizzetti P, Lo Gullo C, et al. Resection of obstructing bronchial fibrolipoma through the flexible fiberoptic bronchoscope. Endoscopy 1982;14:61–63.
5. Pedersen U, Kristensen S, Illum P. Palliative resection with high-frequency cutting loop in malignant tracheobronchial diseases. J Bronchol 1994;1:23–25.
6. Hooper RG, Spratling L, Beechler C, et al. Endobronchial electrocautery: a role in bronchogenic carcinoma? Endoscopy 1994;16:67–70.

